# Assessing the Environmental Impact of Municipal Waste on Energy Incineration Technology for Power Generation Using Life Cycle Assessment Methodology

**DOI:** 10.3390/toxics12110786

**Published:** 2024-10-29

**Authors:** Yiting Luo, Mingqiang Ye, Yihui Zhou, Rongkui Su, Shunhong Huang, Hangqing Wang, Xiangrong Dai

**Affiliations:** 1 Hunan First Normal University, Changsha 410114, China; 2 National Engineering Laboratory of Southern Forestry Ecological Application Technology, Changsha 410004, China; 3 Hunan Provincial Key Laboratory of Key Technology on Hydropower Development, PowerChina Zhongnan Engineering Corporation Limited, Changsha 410004, China; 4 Aerospace Kaitian Environmental Technology Co., Ltd., Changsha 410100, China; 5 College of Life and Environmental Science, Hunan Engineering Research Center of Full Life-Cycle Energy-Efficient Buildings and Environmental Health, Central South University of Forestry and Technology, Changsha 410004, China

**Keywords:** life cycle method, garbage, incineration power generation, energization, environmental impact

## Abstract

The life cycle assessment methodology is a comprehensive environmental impact evaluation approach rooted in the “cradle-to-grave” concept. This study takes a municipal solid waste incineration power plant in central China as an example to comprehensively explore the potential ecological and environmental impacts of municipal solid waste incineration power generation through life cycle assessment methods. Burning one ton of waste can recover 7342 joules of thermal energy. Compared with traditional landfill, incineration can reduce greenhouse gas emissions by about 30%, with a potential global warming impact of −0.69 kg of carbon dioxide equivalent. Amongst environmental impacts, land, freshwater, and marine ecosystems possess the greatest potential toxicity, followed by the harmful effects on human health and the influence of ozone-producing photochemical pollution. Lastly, there comes terrestrial acidification, whereas other types of effects can be relatively disregarded in comparison. In the process of waste incineration power generation, the potential impacts of global warming, ionizing radiation, and fossil resource scarcity are less than zero, indicating that this is an environmentally friendly process. In response to the above-mentioned environmental impacts, it is necessary to pay attention to improving incineration efficiency, optimizing leachate treatment, reducing coal use, and controlling acidic gas emissions in the process of urban solid waste incineration power generation. This research offers insights into advancing environmentally sustainable technologies for utilizing waste as an energy resource.

## 1. Introduction

Waste, including trash and rubbish, represents one of the fastest-growing categories of environmental disposables [1]. It encompasses the solid materials generated through human daily activities, characterized by diversity and complexity [2,3]. Research indicates that, in 2017, China’s nationwide production of food waste reached 99.72 million metric tons, with an annual growth rate exceeding 10% [4]. Over the period from 2005 to 2025, food and kitchen waste across 70 Asian countries and regions is projected to increase from 278 million tons to approximately 416 million tons. Concurrently, per capita daily waste generation rates in urban areas of the European Union and Asia are expected to rise between 0.9 to 1.6 kg and 0.7 to 1.5 kg, respectively [5]. “Urban Waste” refers to the waste generated in urban areas around the world, which includes various types of waste produced by households, commercial establishments, and industrial activities within cities [6]. The waste can consist of organic materials, plastics, metals, paper, textiles, and hazardous substances. Global urban waste is anticipated to escalate from 1.3 billion tons per year to 2.2 billion tons in 2025, and projected to further reach 4.2 billion tons annually by 2050 [7]. Urban waste significantly impacts both human well-being and the environment. In India, urban waste production grows annually by 1.3%, with a substantial 94% of waste being inadequately managed. Similarly, 93.5% of municipal waste is disposed of in landfills or open-air dumps in Malaysia, with no recycling efforts [8]. “Urban solid waste” refers to the solid waste generated in urban areas, including various materials produced by households, commercial institutions, and industrial activities [6]. In 2015, the United States Environmental Protection Agency designated urban solid waste as a potential renewable energy source. Effective waste management remains a critical global challenge for achieving sustainable development goals [9,10,11].

As society develops, more and more attention is paid to environmental protection in the world [12,13,14,15,16]. As the accumulation of urban solid waste continues to rise, the efficient management of such waste in a sustainable and environmentally friendly manner has become the most pressing social and environmental challenge faced by urban residents today [17,18,19]. Currently, waste management techniques primarily encompass the methods of landfilling, incineration, and comprehensive utilization [20,21]. Due to regional disparities in economic and consumption levels, composition of waste materials, and geographical environments, the prevailing conditions for employing these three waste management techniques (landfilling, incineration, and comprehensive utilization) vary, thereby necessitating divergent disposal methods [22]. Overall, the trend indicates that urban solid waste treatment technology has evolved from primitive and arbitrary landfilling to primarily utilizing incineration for power generation [6]. This shift can be attributed primarily to the substantial reduction in available land, along with the heightened awareness of environmental conservation and advances in management expertise among humankind. Consequently, it has gradually facilitated the adoption of waste incineration for energy generation as the predominant mode of waste disposal, emphasizing reduction, harmlessness, and resource recovery [23]. Incineration for power generation is only one manifestation of the technological conversion of waste into energy. Waste-to-energy (WTE) technology, in essence, encompasses the process whereby energy is harnessed in the form of heat, electricity, or even fuel for transportation from sources comprising discarded materials [24,25,26,27]. In the application of WTE technology, municipal solid waste (MSW) is the most prevalent form of energy conversion treatment [28]. The successful application domains of WTE technology encompass industrial green heating fuel granules, the paper and pulp industry’s transformation of waste into cold and thermal electric energy sources, along with urban solid waste treatment plants serving as district energy supply hubs, amongst others [29]. Furthermore, by managing the relatively small proportion of biowaste within the mixed municipal solid waste, not only is the moisture content of the waste reduced but its calorific value is also enhanced [30,31,32,33]. Currently, there are more than 800 waste conversion facilities operating across approximately 40 countries and regions, and they have processed approximately 11% of global municipal solid waste and generated a total power output of 42.9 billion kilowatt-hours. It is estimated that global urban solid waste recycling will generate an energy value of USD 410 billion [34]. The market potential of waste incineration for electricity generation in Chinese urban areas is expected to reach USD 5 billion [35]. Nowadays, waste incineration power generation technology presents a sophisticated solution for energy utilization [36,37], showcasing tremendous prospects for advancement [38,39].

With the rapid development of technology and the gradual improvement in people’s living standards, the inorganic components in waste are gradually decreasing while the organic components are increasing [40,41]. Among them, organic waste has a higher calorific value and is beneficial for waste incineration power generation [42,43]. The main sources of waste incineration power generation are municipal solid waste and other screened waste with high organic components. The good practice is to separate and recycle the waste, which not only reduces the amount of waste but also screens out waste with high organic components. The Solid Waste Pollution Prevention and Control Law of the People’s Republic of China (2020) stipulates that municipal solid waste should be promptly cleared and transported and gradually classified and collected, and that rational utilization and harmless disposal is actively carried out. Plastics, metals, and glass, as recyclables, are found less and less in municipal solid waste, which was used as a source of raw materials for garbage incineration. The incineration treatment of various types of waste in life, followed by their utilization for electricity generation, adheres to the three principles of reduction, resource utilization, and harmlessness [44,45]. High temperature incineration can significantly reduce the amount of waste disposed of in landfills while utilizing the generated thermal energy to generate electricity and reusing the by-products for road construction [46]. The incineration processing facility, being reasonable and sustainable, serves as a viable alternative to landfill for waste disposal, without compromising the rates of reuse and recycling [9]. In the year 1988, China’s inaugural waste incineration plant commenced operation in Shenzhen. Though our nation embarked on waste incineration relatively late, China’s waste incineration-to-electricity technology has made remarkable strides. According to a report by the Asian Development Bank (ADB) in 2007, China has emerged as the second-largest producer of municipal solid waste worldwide. Annually, an excess of 220 million metric tons of such waste is generated, with an annual growth rate ranging between 8% and 10%. In the year 2018, the quantity of waste incineration power plants in China witnessed an increase of 11.9% compared with the previous year (reaching a total count of 401), enabling them to process over 40% of the national waste transportation volume. Furthermore, in the year 2019, an additional 50% increase occurred in nationwide waste incineration-to-electricity initiatives [47]. Compared with the photovoltaic and other industries, China’s currently operational waste incineration power generation projects exhibit relatively low capital investment, CO_2_ emission-friendly characteristics, low costs, and desirable environmental benefits [48,49].

Household waste comprises a complex mixture that generates numerous pollutants during the incineration process, including exhaust emissions, wastewater, noise, and solid residues. These pollutants significantly impact the environment throughout the waste-to-energy incineration process [50]. However, the current research predominantly focuses on technological advancements and efficacy studies related to waste-to-energy incineration, with comparatively less attention given to evaluating its environmental impacts [45,51,52]. The life cycle assessment (LCA) method is a multidimensional environmental impact evaluation approach that follows the concept of “from cradle to grave”. It has substantial capabilities for tracking the evolution of energy products and ensures the credibility and scientific rigor of assessing environmental impacts associated with waste-to-energy conversion [53,54,55]. This study employs the life cycle method to present a specific case study on the energy utilization of waste incineration for power generation. It includes defining objectives and scope, analyzing life cycle inventory, assessing environmental impacts throughout the life cycle, and interpreting life cycle results. Through comprehensive analysis and evaluation, potential environmental risks related to waste incineration power generation are identified, and targeted improvement measures and suggestions are proposed to promote the sustainable development of environmental hazardous waste energy utilization technology.

## 2. Materials and Methods

### 2.1. The Principles of the Life Cycle Methods

As human civilization advances, the escalating degradation of the global ecological environment has become increasingly evident. Conventional methods of managing and addressing environmental issues are no longer adequate for the diverse array of challenges we face today. Therefore, adopting an environmental management approach grounded in life cycle thinking is more conducive to achieving a harmonious and sustainable balance between the economy and the environment [56]. Life cycle assessment (LCA), as an environmental management tool for product systems, has gradually gained acceptance and application across various domains, being recognized as the most promising environmental management tool of the 21st century [57,58]. The International Organization for Standardization (ISO) defines life cycle assessment as the quantitative and qualitative analysis and evaluation of resource consumption and waste emissions attributable to a product throughout its life cycle in its ISO 14040 standard [59].

Therefore, LCA should follow several fundamental principles. First, LCA emphasizes comprehensiveness, requiring that all stages from raw material extraction, production, transportation, and usage to final disposal be considered in the assessment to ensure a holistic understanding of environmental impacts. Second, LCA focuses on quantitative analysis, collecting and analyzing relevant data to quantify resource consumption and emissions at each stage, thereby identifying environmental hotspots. Additionally, LCA emphasizes transparency and reproducibility, requiring that the research process and results be subject to external review and verification. It focuses on midpoint (problem oriented) and endpoint (damage oriented) impact categories. The life cycle approach evaluates the “inputs” and “outputs” of each process [60]. According to ISO14040 (Environmental Management-Life cycle assessment-Principles and framework, International Organization for Standardization, 2006), we will have certain trade-offs in the process of discussion and traceability, as long as the corresponding trade-off rules are met, otherwise the traceability will be endless. The regulations also point out that there are two types of LCA that do not need to trace the process. One is natural resources. we only need to know the consumption of natural resources, and do not need to trace their production process. One is environmental emissions (including greenhouse gases, atmospheric/water/soil pollutants, solid waste, etc.). we only need to discharge, and do not need to know the natural process after discharge. This systematic methodology makes LCA an important tool for supporting sustainable development decisions, providing scientific evidence for businesses and policymakers to optimize product design and improve environmental management strategies.

### 2.2. The Fundamental Analytical Framework of the Life Cycle Approach

This study adheres to the boundary demarcation and data processing standards outlined in ISO 14040 and GB/T 24040 (Environmental management. Life cycle assessment. Principles and frameworks, China National Standards Committee, 2008). We utilize the ReCiPe 2016 Midpoint (I) V1.04/World (2010) I for calculations, which is widely employed in life cycle assessment (LCA) to analyze and quantify the environmental impacts of a product across its entire life cycle, from raw material acquisition through production, use, and disposal [61]. According to ISO14040 and GB/24040, the entire process of life cycle assessment can be divided into four main components, namely the definition of objectives and scope, analysis of the life cycle inventory, evaluation of the life cycle impacts, and interpretation of the life cycle results [59,62,63]. The relationship between these components is depicted in our previous reports [64].

## 3. Results and Discussion

### 3.1. Basic Framework of the LCA for Waste Incineration Power Generation

(1) Definition of objectives and scope

The four steps of implementing a life cycle assessment are used to evaluate the LCA process of waste incineration in the living environment, and to study the environmental impact of waste heat and flue gas cleaning processes during incineration. Previous studies on the life cycle assessment of urban solid waste incineration have mainly focused on technological innovations in waste incineration processes, with few reports on the specific impacts during the incineration stages. This study aims to assess the environmental impact generated from waste-to-energy processes, and proposes benchmarks for preventing and reducing pollution from waste incineration. Therefore, various comparative methods are determined to define the scope of this research system, including pretreatment, post-incineration treatment, and emissions. This case selects a waste incineration power plant in central China, with a designed processing capacity of 1000 tons/day, equipped with 2 sets of 500 t/d mechanical grate incinerators and a 18 MW condensing steam turbine generator units. The annual operating hours are 8000 h, the annual waste processing capacity is 365,000 tons, and the annual power generation is 136.3 million kWh. Simultaneously supporting the construction of environmental protection projects such as flue gas purification systems, wastewater treatment systems, and ash treatment systems, garbage incineration power plants implement the “Control Standard for Pollutants from Municipal Solid Waste Incineration” (GB 18485-2014, China National Standards Committee). According to the requirements of the “Control Standard for Pollutants from Municipal Solid Waste Incineration” (GB 18485-2014, China National Standards Committee), the incineration temperature in the furnace is greater than or equal to 850 degrees Celsius, the residence time of flue gas in the furnace is greater than or equal to 2 s, and the thermal burn off rate of incinerator slag is less than or equal to 5%. In this study, the functional unit for generating electricity from waste incineration is 1 ton of waste.

**Figure 1:**
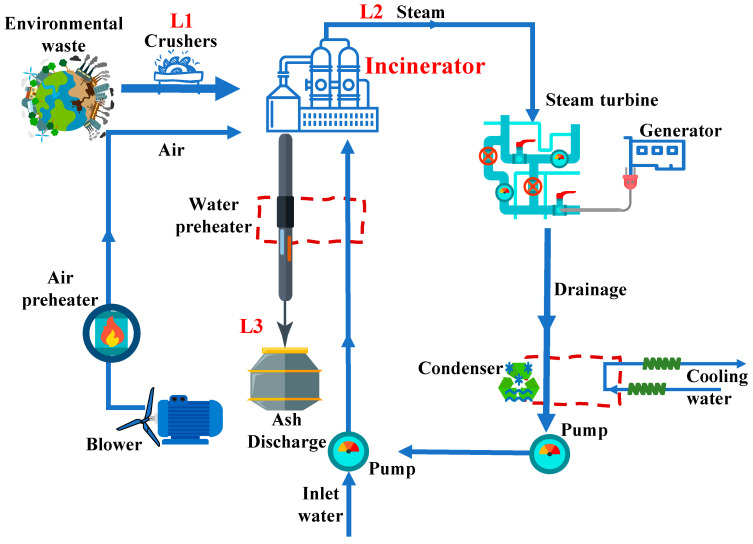
Diagram of environmental waste incineration power generation process. Output points for testing purposes (L1, L2, L3).

**Figure 2:**
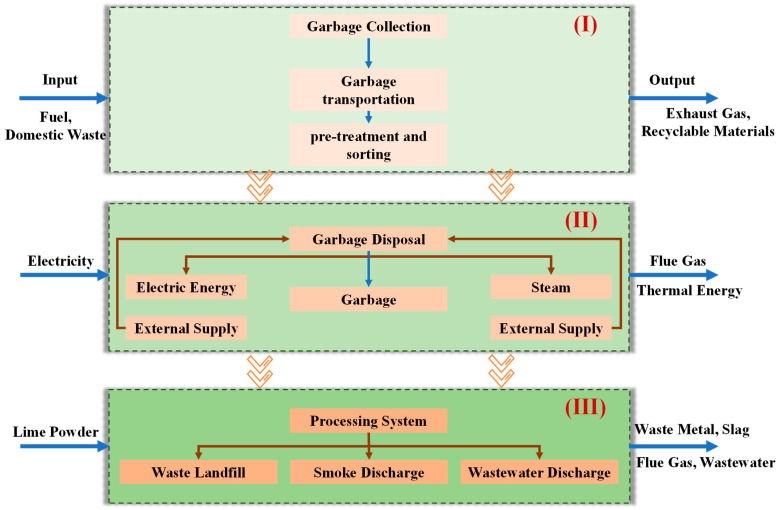
Illustration of boundary demarcation, the pre-processing stage (**I**), the incineration stage (**II**), and the exhaust emission stage (**III**).

The model calculates the emissions and resource consumption of air, water, and soil, and consolidates the results into various potential impacts. Based on the ReCiPe model and its database, the composition of solid waste can be characterized by using 48 different material compositions, and each component is defined by 40 physical and chemical properties, including parameters such as the low heating value (LHV) of the fuel, moisture content, ash content, and elemental composition. Additionally, a comprehensive life cycle inventory (LCI) database is provided to facilitate energy generation and material production from a wide array of fuels. The energy produced by the incineration system and the recovered materials replace conventional fuel combustion and material manufacturing derived from raw resources. Consequently, emissions associated with air, water, soil, and resource consumption are mitigated through the incineration process. The schematic diagrams of the waste incineration process and boundary delineation are illustrated in Figure 1 and Figure 2, respectively. Considering the intricate nature of the incineration system, it has been segregated into three distinct segments for analytical purposes. The sampling analysis shall be conducted on the L1, L2, and L3 locations within these three systems, enabling a comprehensive evaluation of the environmental factors associated with each subsystem.

(2) Inventory Analysis

The analysis of the data inventory, consistent with the aforementioned study, primarily focuses on organizing and analyzing the energy processes within defined research system boundaries. It also aims to assess the environmental potentials associated with activities such as the extraction of raw materials, processing, product manufacturing, packaging and transportation, consumption, and waste management. To quantitatively and rationally analyze the consumption of resources, energy, and environmental emissions, it is imperative to develop a comprehensive inventory known as the input–output table. The data presented in this section are derived from extensive research of the literature. Refer to Table 1 for details.

### 3.2. Assessment of the Environmental Impacts of Incinerating Waste for Power Generation

(1) Identification of types of environmental impact

Due to the intricate nature of the waste incineration process for generating electricity, it is divided into three stages for analysis: the pre-processing stage (I), the incineration stage (II), and the exhaust emission stage (III). Given that the efficiency of the air purifier is limited, significant emission gases such as SO_2_, CO_2_, CO from incineration, NO_X_ generated by engine operation, and other VOCs are selected as output points for testing purposes (L1, L2, L3). Five tests are conducted to ensure data reliability, with an average value obtained. Specific data can be found in Table 2, Table 3 and Table 4.

Table 2, Table 3 and Table 4 provide a detailed description of the gas emission data measured at different monitoring points (L1, L2, and L3) during the process of waste incineration for power generation. Table 2 lists the gas emission concentrations at point L1, where the average concentration of CO_2_ is 499.2 mg/m^3^, the average concentration of SO_2_ is 10.4 mg/m^3^, and the concentration of NO is 0.066 mg/m^3^. Table 3 focuses on the emission situation at point L2, showing that the concentration of carbon dioxide is 35,532.2 mg/m^3^, sulfur dioxide is 108 mg/m^3^, and nitrogen monoxide is 0.876 mg/m^3^. Finally, Table 4 provides the emission data for point L3, with a carbon dioxide concentration of 9532.2 mg/m^3^, sulfur dioxide of 34.4 mg/m^3^, and nitrogen monoxide of 0.582 mg/m^3^. These data, obtained through multiple measurements, provide a quantitative basis for assessing the environmental impact of gas emissions during the incineration process, revealing the emission characteristics at different monitoring points and their potential environmental risks, emphasizing the effectiveness of incineration technology in emission reduction and its importance for environmental management.

(2) Calculation of standardized values for environmental impact

In life cycle assessment (LCA), characterization factors refer to the coefficients used to quantify specific environmental impacts. These factors are typically used to convert the emissions of substances (such as greenhouse gas emissions, releases of toxic substances, etc.) into the potential impact amounts of specific environmental impacts, such as global warming potential (GWP), acidification potential (AP), or ozone depletion potential (ODP). Employing the ReCiPe model [61], we calculate the characterization factors of the features to shed light on distinct types of environmental impacts. The purpose of characterizing calculations is to convert different substances from each type of environmental impact into a unified parameter for subsequent weighted calculations. In relation to global hot environmental issues, this paper primarily focuses on 18 types of environmental impacts during the process of waste incineration for electricity generation. These specifically include, global warming, stratospheric ozone depletion, ionizing radiation, ozone formation and human health impacts, fine particulate matter formation, terrestrial acidification, ozone formation in terrestrial ecosystems, freshwater eutrophication, marine eutrophication, terrestrial ecotoxicity, marine ecotoxicity, freshwater ecotoxicity, human carcinogenic toxicity, human non-carcinogenic toxicity, scarcity of mineral resources, scarcity of fossil resources, and environmental impacts related to land use and water consumption. ReCiPe 2016 has already provided a detailed description of the impact categories and characteristic factors. The specific standardization results are shown in Table 5. The characterizing calculation results are obtained by multiplying the corresponding gas emissions with their respective relevant factors to obtain the final outcome [72].

(3) Normalization and weighted calculations

After computing the characteristic values, it is necessary to standardize and assign weights to the obtained impact potentials. In this study, the environmental impact load of 1990 was taken as the benchmark value for weighted calculations, with a step value of 1. The formula for calculating standardized potential values of environmental pollutants’ effects is provided below [73,74]:

(1) $${\mathrm{NPE}}_{m}={\mathrm{EP}}_{m}/{\mathrm{ER}}_{m}$$

In the provided equation, NPE*_m_* represents the normalized data value of the *m*-th type of environmental impact potential; EP*_m_* denotes the environmental potential value for the *m*-th type of environmental impact within the system; and ER*_m_* corresponds to the standardized data value of the *m*-th type of environmental impact potential. The computational outcome is depicted in Table 6.

### 3.3. Analysis of the Environmental Impacts and Improvement Measures for Incineration of Industrial Waste

Upon examining Table 5 and Table 6, it can be inferred that during the process of waste incineration for power generation, the potential impacts of global warming, ionizing radiation, and the scarcity of fossil resources are negative, indicating a type that is environmentally friendly. Greenhouse gases generated by waste incineration are offset by the energy recovery benefits generated by recycling, while also resolving the issue of depletion in other fossil resources. Furthermore, if waste is not adequately addressed through incineration, the significant impact of ionizing radiation resulting from its storage or landfilling persists; however, such impacts are eliminated after undergoing incineration. Amongst environmental impacts, land, freshwater, and marine ecosystems possess the greatest potential toxicity, followed by the harmful effects on human health and the influence of ozone-producing photochemical pollution. Lastly, there comes terrestrial acidification, whereas other types of effects can be relatively disregarded in comparison. Due to the intricate composition of various discarded materials, during the incineration process, a wide range of toxic and harmful gases, waste residues, and leachate pollutants unavoidably lead to an ecological toxicity evolution, thereby resulting in ecological imbalance. Furthermore, the incomplete combustion of gases such as CO and CH_4_ during the incineration process exacerbates the formation of ozone, leading to photochemical pollution. In addition, other acidic gases such as H_2_S and SO_2_ are emitted into the atmosphere, causing air and land acidification. Ultimately, these factors significantly impede humanity and its way of life. Therefore, further analysis is conducted on the aforementioned prominent potentialities of detrimental impacts, pertaining to the four distinct types of environmental influences. Corresponding enhancements are subsequently proposed in relation to these four influences. Based on the aforementioned computational analysis, it can be inferred that, within the process of utilizing incineration for generating electricity from environmental waste, the primary environmental factor with the most profound impact is ecological toxicity, followed by human toxicity hazards. Lastly, there are concerns surrounding photochemical pollution caused by ozone formation and environmental acidification. With regard to these categories, there are proposed measures for improvement as follows:

(1) In order to effectively reduce the ecological and human toxicity caused by the emission of harmful substances, such as dioxin gas and fly ash, we can enhance the incineration efficiency to ensure full decomposition and minimize emission concentrations. As for common solid waste like furnace slag, it can typically be directly utilized as a secondary material.
(2) Leachate from waste infiltration will penetrate groundwater and surface water sources due to gravitational runoff, causing the contamination of natural water sources and downstream soil. Therefore, the treatment and prevention of leachate are of the utmost importance. For instance, high temperature oxidative decomposition can be carried out on the percolate through an incinerator for effective disposal.
(3) The primary culprit responsible for ozone is carbon monoxide gas. Therefore, it is imperative to minimize the consumption of coal during combustion. In addition, enhancing the efficiency of combustion calls for the introduction of novel techniques and advanced equipment, which will ensure a more thorough incineration of environmental waste while simultaneously reducing emissions and input requirements.
(4) Environmental acidification primarily occurs due to the presence of acidic gases, such as hydrogen sulfide and ammonia, which are irritants in nature. These gases are generated at various stages of processing. Consequently, resolving the issue pertaining to these gases becomes a matter of utmost importance. Therefore, when handling and unloading waste, it may be advisable to install air curtains at the entrances and exits to prevent the escape of harmful gases. Additionally, during waste storage, periodic movement or agitation should be conducted to prevent fermentation and gas production. Furthermore, it is imperative to enhance the efficiency of smoke purification equipment, directing any unburned hazardous gases through ducts and fans into adsorption and filtration devices for treatment before achieving compliance with emission standards.
(5) Traffic conditions have a significant impact on the site selection of waste incineration power plants through energy consumption and exhaust emissions. The location of a waste incineration power plant should be as close as possible to the service area and the slag/fly ash processing area, and the transportation distance should be economically reasonable. There should be good traffic conditions between the waste incineration power plant, the service area, and the slag/fly ash disposal area.

## 4. Conclusions

This study employs a life cycle assessment (LCA) approach to conduct a comprehensive and detailed evaluation and analysis of the environmental impacts of the waste incineration power generation process. The results reveal the potential environmental impacts of each stage of the incineration power generation process and highlight key environmental issues through quantitative data. During the waste collection, transportation, and incineration stages, this study identifies multiple environmental impact factors and proposes benchmarks for preventing and reducing incineration pollution through model assessments.

The environmental impact assessment of the incineration process indicates that while waste-to-energy incineration has positive effects in reducing global warming, ionizing radiation, and fossil resource scarcity, it also poses significant environmental risks, particularly concerning ecological toxicity, human health hazards, ozone formation, and land acidification. The gas emission data measured at different monitoring points during the incineration power generation process provide quantitative evidence for assessing the environmental impact of gas emissions. The characterization and normalization calculations of environmental impacts show that the global warming potential value is −0.07 kg CO_2_ eq, indicating that incineration power generation contributes to reducing greenhouse gas emissions. However, the impact potential for terrestrial, freshwater, and marine ecological toxicity is the highest, followed by the toxicity hazard to human health, quantified at 435.72 kg 1,4-DC eq. The impacts of photochemical pollution from ozone formation and land acidification are relatively minor.

Additionally, the overall environmental impact of waste energy recovery is predicted. Based on model data analysis, it can be inferred that the environmental impacts during the incineration power generation process are primarily beneficial to the environment, with a focus on global warming, fossil resource scarcity, and ionizing radiation. These factors provide positive benefits to the environment. Furthermore, the ranking of potential environmental impacts is as follows: ecological toxicity > potential human toxicity > photochemical ozone formation > environmental acidification. Air pollutants generated during this process, such as PM_2.5_, SO_2_, CO, HCl, dioxins, and H_2_S, have significant impacts on the environment, necessitating concentrated prevention and control efforts.

In response to the aforementioned harmful impacts, this study proposes a series of improvement measures, including enhancing incineration efficiency, optimizing leachate treatment, reducing coal usage, and controlling acidic gas emissions. These measures aim to reduce the toxic substance emissions that harm ecology and human health, minimize leachate pollution to water sources and soil, improve combustion efficiency to reduce emissions of gases like CO and CH_4_, and control acidic gas emissions to mitigate environmental acidification.

## Figures and Tables

**Table 1:** Burning system input and output.

Burning System	Import		Export	
	Parameter	Magnitude	Parameter	Magnitude
**I**	Waste collection/kg·(people·a)^−1^	670	The amount of recyclable produced [65]/kg·t^−1^	121
	Car fuel usage [66]/L·km^−1^	0.31	Automobile exhaust gas production volume [67]/kg·L^−1^	3.675
**II**	Electric power consumption [68,69]/kwh·(people·a)^−1^	134	Flue gas emissions [70]/kg·t^−1^	632.23
			Thermal energy [65]/J·g^−1^	7342
**III**	Lime powder consumption [67]/kg·t^−1^	1.71	The amount of waste metal produced [71]/kg·t^−1^	30.0
			The amount of flue gas after purification [70]/kg·t^−1^	121.41
			Wastewater discharge [71]/m^3^·t^−1^	89.0
			Amount of slag produced after treatment [67]/kg·t^−1^	270

**Table 2:** Detection of flue gas emissions from the pre-processing stage of environmental waste at L1 monitoring station.

Project	Detection 1	Detection 2	Detection 3	Detection 4	Detection 5	Average Value
Carbon dioxide (CO_2_) g·m^−3^	558	428	572	521	417	499.2
Sulfur dioxide (SO_2_) g·m^−3^	12	10	10	11	9	10.4
Nitrous oxide (N_2_O) g·m^−3^	0	0	0	0	0	0
Carbon monoxide (CO)g·m^−3^	0.05	0.08	0.06	0.07	0.07	0.066
Nitric oxide (NO_X_) g·m^−3^	56	67	65	53	62	60.6
VOC g·m^−3^	0	0	0	0	0	0

**Table 3:** Detection of flue gas emissions from the incineration process of environmental waste at L2 monitoring station.

Project	Detection 1	Detection 2	Detection 3	Detection 4	Detection 5	Average Value
Carbon dioxide (CO_2_) g·m^−3^	35,423	36,428	34,872	35,621	35,317	35,532.2
Sulfur dioxide (SO_2_) g·m^−3^	100	98	100	110	102	108
Nitrous oxide (N_2_O) g·m^−3^	0.22	0.31	0.26	0.29	0.32	0.28
Carbon monoxide (CO) g·m^−3^	0.87	0.89	0.87	0.90	0.85	0.876
Nitrogen oxide (NO_X_) g·m^−3^	856	897	885	843	892	874.6
VOC g·m^−3^	0.52	0.49	0.56	0.58	0.55	0.54

**Table 4:** Detection of flue gas emissions from the exhaust emission stage of environmental waste at L3 monitoring station.

Project	Detection 1	Detection 2	Detection 3	Detection 4	Detection 5	Average Value
Carbon dioxide (CO_2_) g·m^−3^	9423	9428	9872	9621	9317	9532.2
Sulfur dioxide (SO_2_) g·m^−3^	32	36	40	31	33	34.4
Nitrous oxide (N_2_O) g·m^−3^	0	0	0	0	0	0
Carbon monoxide (CO) g·m^−3^	0.55	0.58	0.60	0.57	0.61	0.582
Nitrogen oxide (NO_X_) g·m^−3^	105	97	110	108	98	103.6
VOC g·m^−3^	0	0	0	0	0	0

**Table 5:** Characterization of the environmental effects.

Project	Unit	Altogether	Wastewater Solid Waste Discharge	Slaked Lime	Acticarbon	Process Water	Electricity	Compressed Gas	Ammonia	Fire Coal	Energy Recovery
Global warming	kg CO_2_ eq	−732.78	3472.22	1.75	6.85	498.28	11.59	315.043	0.96	2390.99	−7430.47
Stratospheric ozone depletion	kg CFC11 eq	−0.00141	0	5.18 × 10^−7^	1.04 × 10^−6^	3.37 × 10^−4^	3.16 × 10^−6^	7.55 × 10^−5^	3.62 × 10^−7^	7.80 × 10^−5^	−0.002
Ionizing radiation	kBq Co-60 eq	−42.22	0	0.07	0.21	64.06	0.16	45.72	0.02	4.07	−156.54
Ozone formation and human health	kg NO_x_ eq	1806.10	1811.11	0.004	0.01	1.02	0.03	0.53	0.001	1.77	−8.38
Fine particulate matter formation	kg PM_2.5_ eq	1.63	0	0.001	0.002	0.54	0.004	0.41	0.0002	1.39	−0.72
Ozone formation and the terrestrial ecosystems	kg NO_x_ eq	1805.93	1811.11	0.004	0.01	1.04	0.03	0.54	0.001	1.78	−8.58
Land acidification	kg SO_2_ eq	1158.61	1168.66	0.006	0.03	1.60	0.07	1.09	0.002	2.84	−15.69
Freshwater eutrophication	kg P eq	0.56	0	0.0004	0.002	0.26	0.001	0.16	6.32	0.83	−0.68
Ocean eutrophication	kg N eq	0.04	0	0.0001	0.0001	0.03	8.12	0.01	4.23	0.05	−0.05
Land ecological toxicity	kg 1,4-DC eq	305,617.08	309,375	1.44	2.17	455.23	3.04	97.07	2.25	207.51	−4526.63
Freshwater ecological toxicity	kg 1,4-DC eq	21.98	0.35	0.03	0.10	19.19	0.21	5.68	0.02	30.26	−33.88
Marine ecological toxicity	kg 1,4-DC eq	33.80	29.33	0.008	0.03	5.95	0.05	1.78	0.007	10.00	−13.38
Human oncogenic toxicity	kg 1,4-DC eq	128.02	128.83	0.0005	0.002	0.27	0.001	0.07	0.0004	0.47	−1.62
Non-carcinogenic toxicity in humans	kg 1,4-DC eq	13,531.63	13,536.50	0.02	0.13	11.44	0.06	3.02	0.01	20.16	−39.80
Land use	m^2^a crop eq	31.14	0	0.37	0.70	76.68	0.08	6.42	0.04	169.0	−222.17
Mineral resources are scarce	kg Cu eq	2.59	0	0.002	0.002	3.54	0.004	0.23	0.002	0.63	−1.82
Fossil resources are scarce	kg oil eq	−1205.92	0	0.35	1.52	113.62	1.56	64.69	0.38	924.06	−2312.04
Water consumption	m^3^ eq	652.57	0	0.06	0.02	558.45	0.04	96.71	0.03	5.37	−8.10

**Table 6:** Normalized characterization factors.

Project	Altogether	Wastewater Solid Waste Discharge	Slaked Lime	Acticarbon	Process Water	Electricity	Compressed Gas	Ammonia	Fire Coal	Energy Recovery
Global warming	−0.07	0.32	0.0001	6.0 × 10^−5^	0.05	0.001	0.03	8.92 × 10^−5^	0.22	−0.69
Stratospheric ozone depletion	−0.02	0	7.9 × 10^−6^	1.6 × 10^−5^	0.005	4.8 × 10^−5^	0.001	5.5 × 10^−6^	0.001	−0.03
Ionizing radiation	−0.08	0	0.0001	0.0004	0.14	0.0003	0.10	3.5 × 10^−5^	0.008	−0.33
Ozone formation and human health	87.78	88.02	0.0002	6.0 × 10^−5^	0.05	0.001	0.03	5.1 × 10^−5^	0.09	−0.41
Fine particulate matter formation	0.10	0	9.0 × 10^−5^	1.0 × 10^−5^	0.03	0.0003	0.03	1.6 × 10^−5^	0.09	−0.05
Ozone formation and the terrestrial ecosystems	101.67	101.97	0.0003	7.0 × 10^−5^	0.06	0.001	0.03	6.1 × 10^−5^	0.10	−0.48
Land acidification	28.27	28.52	0.0001	7.0 × 10^−5^	0.04	0.001	0.03	5.6 × 10^−5^	0.07	−0.38
Freshwater eutrophication	0.87	0	0.0006	0.003	0.40	0.001	0.24	9.7 × 10^−5^	1.27	−1.04
Ocean eutrophication	0.009	0	2.3 × 10^−5^	3.5 × 10^−5^	0.005	1.7 × 10^−5^	0.002	9.1 × 10^−7^	0.01	−0.01
Land ecological toxicity	550.11	556.88	0.002	0.003	0.82	0.005	0.17	0.004	0.37	−8.15
Freshwater ecological toxicity	21.63	0.35	0.03	0.10	18.89	0.20	5.59	0.02	29.78	−33.33
Marine ecological toxicity	85.163	73.92	0.02	0.08	15.01	0.13	4.48	0.02	25.21	−33.71
Human cancer	54.92	55.27	0.0002	0.0008	0.12	0.0005	0.03	0.0002	0.20	−0.69
Human non-carcinogenic	435.72	435.88	0.0007	0.004	0.37	0.001	0.10	0.0003	0.65	−1.28
Land use	0.005	0	5.9 × 10^−5^	0.0001	0.012	1.3 × 10^−5^	0.001	6.5 × 10^−6^	0.03	−0.04
The scarcity of mineral resources	1.30 × 10^−5^	0	1.2 × 10^−8^	1.2 × 10^−8^	1.8 × 10^−5^	2.2 × 10^−8^	1.1 × 10^−6^	1.0 × 10^−8^	3.2 × 10^−6^	−9.4 × 10^−6^
Fossil resources are scarce	−1.23	0	0.0003	0.001	0.12	0.001	0.07	0.0003	0.94	−2.36
Water consumption	2.45	0	0.0002	6.10 × 10^−5^	2.09	0.0001	0.36	9.40 × 10^−5^	0.02	−0.03

## Data Availability

Data are contained within the article.
